# Leptin-dependent neurotoxicity via induction of apoptosis in adult rat neurogenic cells

**DOI:** 10.3389/fncel.2015.00350

**Published:** 2015-09-07

**Authors:** Stéphanie Segura, Laurie Efthimiadi, Christophe Porcher, Sandrine Courtes, Valérie Coronas, Slavica Krantic, Emmanuel Moyse

**Affiliations:** ^1^Physiologie de la Reproduction et des Comportements, UMR 85 Institut National de la Recherche Agronomique, Centre INRA de Tours, Université François Rabelais de ToursNouzilly, France; ^2^Institut National de la Santé et de la Recherche Médicale Unité 901, Institut de Neurobiologie de la Méditerranée, Parc Scientifique de Luminy, Aix-Marseille UniversitéMarseille, France; ^3^Signalisation et Transports Ioniques Membranaires, ERL 7368 Centre National de la Recherche Scientifique, Université de PoitiersPoitiers, France; ^4^Centre de Recherche des Cordeliers, UMR_S 1138 INSERM, Paris Descartes University, Sorbonne Paris Cité, Pierre and Marie Curie UniversityParis, France

**Keywords:** leptin, neurospheres, adult neural stem cells, proliferation, neuronal death, STAT3, ERK, rat

## Abstract

Adipocyte-derived hormone leptin has been recently implicated in the control of neuronal plasticity. To explore whether modulation of adult neurogenesis may contribute to leptin control of neuronal plasticity, we used the neurosphere assay of neural stem cells derived from the adult rat subventricular zone (SVZ). Endogenous expression of specific leptin receptor (ObRb) transcripts, as revealed by RT-PCR, is associated with activation of both ERK and STAT-3 pathways via phosphorylation of the critical ERK/STAT-3 amino acid residues upon addition of leptin to neurospheres. Furthermore, leptin triggered withdrawal of neural stem cells from the cell cycle as monitored by Ki67 labeling. This effect was blocked by pharmacological inhibition of ERK activation thus demonstrating that ERK mediates leptin effects on neural stem cell expansion. Leptin-dependent withdrawal of neural stem cells from the cell cycle was associated with increased apoptosis, as detected by TUNEL, which was preceded by cyclin D1 induction. Cyclin D1 was indeed extensively colocalized with TUNEL-positive, apoptotic nuclei. Cyclin-D1 silencing by specific shRNA prevented leptin-induced decrease of the cell number per neurosphere thus pointing to the causal relationship between leptin actions on apoptosis and cyclin D1 induction. Leptin target cells in SVZ neurospheres were identified by double TUNEL/phenotypic marker immunocytofluorescence as differentiating neurons mostly. The inhibition of neural stem cell expansion via ERK/cyclin D1-triggered apoptosis defines novel biological action of leptin which may be involved in adiposity-dependent neurotoxicity.

## Introduction

Leptin has been identified as a fat storage-reducing hormone in adipose tissue (Zhang et al., 1994). In adult mammals, it is secreted by adipocytes proportionally to their lipidic charge and negatively regulates energy homeostasis (Oswal and Yeo, 2010). Leptin also exerts direct long-term inhibition of feeding behavior at the central level by regulating the key centres of food intake, including hypothalamus (Grill and Hayes, 2012; Morton et al., 2014). These leptin actions are mediated by specific membrane receptors (ObRb; Villanueva and Myers, 2008) that are widely distributed in the adult brain (Elmquist et al., 1998; Myers et al., 2009). Activation of ObRb by leptin binding triggers multiple signaling pathways: (i) Signal Transducer and Activator of Transcription 3 (STAT3), (ii) mitogen-activated protein kinase (MAPK)/extracellular signal-regulated kinase (ERK), (iii) phosphatidylinositol 3-kinase (PI3K)-AKT cascades, (iv) STAT5, (v) 5′ adenosine monophosphate-activated protein kinase (AMPK; Villanueva and Myers, 2008; Coppari and Bjørbæk, 2012).

At the cellular level, leptin regulates structural plasticity of the neuronal networks in rodent hypothalamus, both during development (Bouret et al., 2004; Bouret, 2013) and in adult (Pinto et al., 2004). Among the mechanisms regulating neuronal network plasticity inherent to physiological adaptations, neurogenesis has been established to play an important role (Braun and Jessberger, 2014). Notably, the adult neurogenesis located to the neurogenic niche of rodent hypothalamus (Cheng, 2013; Hann et al., 2013), has been recently involved in food intake regulation (Kokoeva et al., 2005; Pierce and Xu, 2010; Lee et al., 2012; McNay et al., 2012). In addition, leptin has been shown to stimulate adult neurogenesis in murine hypothalamus *in vivo* via expansion of hypothalamic neural stem cells in the context of energy homeostasis and feeding (McNay et al., 2012; Bless et al., 2014).

Food intake regulation is also determined by olfactory perception and memory which is shaped by adult neurogenesis in olfactory bulb (Gheusi and Lledo, 2014) and is modulated by leptin (Palouzier-Paulignan et al., 2012). The exclusive source of olfactory bulb adult neurogenesis is the neural stem cell niche of the subventricular zone of the telencephalon (SVZ) (Braun and Jessberger, 2014). However, SVZ has not been investigated so far in terms of possible regulation of adult neurogenesis by leptin. In the present study, we therefore asked whether leptin regulates adult neurogenesis in the SVZ. To address this question, we used the *in vitro* culture system known as the neurosphere assay (Louis et al., 2013) and analyzed leptin effects on neurospheres derived from adult rat SVZ.

## Materials and methods

### Animals

Forty adult male Wistar rats (ICO: OFA-S.D. [IOPS.Caw]; Charles River, Les Oncins, France), weighing 150–200 g, were used in this study. These animals were bred and handled in accordance with the Guide for the Care and Use of Laboratory Animals (National Research Council, 1996) and the European Communities Council Directive of 24 November 1986 (86/609/EEC). The experimental protocols were carried out in compliance with institutional Ethical Committee guidelines for animal research. All efforts were made to minimize the number of animals used and their suffering.

### Primary culture of neural stem cells

The “neurosphere assay” was performed as previously described (Charrier et al., 2006; Louis et al., 2013). Brains were obtained from adult rats anesthesized and euthanized by decapitation. 500 μm-thick coronal forebrain slices were rapidly cut with a tissue-chopper at the level of anterior striatum, and transferred into ice-cold low-calcium artificial cerebrospinal fluid (aCSF: 124 mM NaCl, 5 mM KCl, 3.2 mM MgCl2, 0.1 mM CaCl2, 26 mM NaHCO3, 100 mM glucose, pH 7.38) for microdissection of the SVZ under binoculars. The tissue samples were digested in 10 U activated papain (Sigma, L'Isle d'Abeau, France) and then by 1X TrypLe™ Express (Invitrogen, Cergy-Pontoise, France), each for 8 min at 37°C, while being triturated gently with a pipet cone. The resulting cell suspension was diluted with 800 μL of culture medium (DMEM [Sigma], 20 μM HEPES [Invitrogen], 200 U/mL penicilline and 200 μg/mL streptomycine [Invitrogen], 1X B27 [Invitrogen, Cergy Pontoise, France], 20 ng/mL basic Fibroblast Growth Factor (bFGF) [Invitrogen], 8 or 20 ng/mL Epidermal Growth Factor (EGF) [Invitrogen]). The cell suspension was then centrifuged at 400 × g for 8 min, the pellet was resuspended in 500 μL of culture medium and triturated with a 1 mL 26G syringe. The cells were seeded at 10,000 cells per 1 mL culture medium per well (24-well plates for non-adherent cells [Corning, Avon, France]) with or without murine recombinant leptin [Amgen, Thousand Oaks, CA, USA]). For passaging, the neurospheres were pooled in a tube and incubated for 30 min at 37°C in 1 mL TrypLe™ Express (Invitrogen). The cell suspension was then diluted with 800 μL of culture medium and centrifuged. The resulting pellet was dissociated, the cell density was counted and adjusted as above. Cell culture medium was changed every 2 days and, when relevant, leptin was added daily.

### Cytochemical assays

For cytochemical assays, primary cultures of adult rat SVZ EGF were grown in the presence of 8 nM during 5 DIV on poly-D-lysine (Sigma)-coated glass coverslips (inserted at the bottom of the 24-well plates) in the absence (control) or in the presence of leptin at the physiologically relevant dose 6.2 nM (Bariohay et al., 2005), rinsed in PBS, fixed 30 min at 4°C with a 4% paraformaldehyde solution in 0.05 M sodium phosphate buffer (pH 7.4), rinsed three times in PBS, and permeabilized in PBS containing 0.1% Triton X-100 and 1% BSA. For immunocytochemistry, primary antibodies (list in Table 1) were incubated in PBS containing 0.1% Triton X-100, 1% BSA, 1% normal serum overnight at 4°C, revealed with relevant Alexa-fluorescent secondary antibodies, counterstained with diamidino-2-phenylindole (DAPI) and mounted on glass slides with Vectashield (Vector labs). *In situ* labeling of apoptotic nuclear DNA fragmentation (TUNEL assay) was performed as previously described (Bauer et al., 2003) for initial quantification of leptin effects. Briefly, the coverslips were incubated 15 min at room temperature with proteinase K at 20 μg/mL in PBS, then 10 min with 2% H_2_O_2_ in PBS, 5 min in Tris-cacodylate-CoCl_2_ (300:140:1 mM) buffer (pH 7.5) and 2 h at 37°C in the same buffer with 150 U/mL terminal-transferase (TdT, Roche-Diagnostics, Meylan, France) and 6 nM biotinylated d-UTP (Roche), rinsed 15 min at room temperature in SSC 1X, and revealed with Alexa-fluorescent avidin (Molecular Probes). For the phenotypic identification of apoptotic cells, a proteinase-K-free fluorescent detection kit was used (Roche Diagnostic, Meylan, France) following the manufacturer's instructions, prior to subsequent immunocytofluorescent labeling of neuropoiesis stage markers (Table 1).

**Table 1:** **List of primary antibodies and details of immunohistochemical procedures used**.

**Antigen**	**Source**	**Cell-type specificity**	**Dilution**
Nestin	Mouse monoclonal, Millipore	Neural stem cells	1:300
Sox-2	Goat polyclonal, Santa-Cruz	Neural stem cells	1:100
Doublecortin (DCX)	Goat polyclonal, Santa-Cruz	Immature/migrating neurons	1:200
Ki-67	Mouse monoclonal, BD Biosciences	Proliferating cells in cell cycle	1:1.000
Cyclin D1	Rabbit monoclonal, Neomarker	Cycling cells in G1/S phases	1:500
Glial fibrillary acidic protein (GFAP)	Rabbit polyclonal, Dako	Astrocytes, neural stem cells, radial glia	1:500
S-100-β	Rabbit polyclonal, Dako, Z0311	Astrocytes	1:400
Oligodendrocytic protein O4	Rabbit polyclonal, Chemicon	Oligodendrocytes	1:75
Microtubule-associated protein-2 (MAP2)	Mouse monoclonal, Sigma-Aldrich	Mature neurons	1:300–1:500
Axonal βIII-tubulin	Mouse monoclonal, Sigma-Aldrich	Mature neurons	1:150

### mRNA extraction and RT-PCRs

Neurospheres (obtained from two rats) were collected after 5–10 DIV and centrifuged 8 min at 400 g. The resulting pellet was subjected to TRIzol (Invitrogen) RNA extraction according to the manufacturer's instructions. RNA was then reverse-transcribed using the Transcriptor First Strand cDNA Synthesis Kit (Roche Applied Science, Mannheim, Germany) with 2.5 μM anchored-oligo(dT)18 primer and 60 μM random hexamer primer in a 20 μl final volume, according to the manufacturer's instructions. PCR amplification was performed using Taq polymerase (Sigma, 1 unit per μL cDNA template) with forward primer 5′-AGTTGTGGTGAAATCACATTGG-3′ and reverse primer 5′-GATATTTGGTCCTCTTCTTCTGG-3′, i.e., the Blast-derived rat homolog of the primer pair that we had used previously for rat ObRb (Fombonne et al., 2007) generating a ObR-specific 438-bp DNA fragment; after 3 min denaturation at 96°C, 33 cycles of 45 s denaturation at 96°C, 30 s annealing at 58°C, 90 s extension at 72°C, and a final 10 min extension at 72°C, amplicons were subjected to electrophoresis on 1% agarose gel pre-stained with ethidium bromide. For quantitative real-time RT-PCR (qPCR), the primers used were Ccnd1 (QT00185241) generating a cyclin D1-specific 109-bp DNA fragment (GenBank NM_022267) and QT00199640 generating an HPRT-specific125-bp DNA fragment (GenBank NM_012583). qPCR was carried out with the LightCycler 480 SYBR Green I Master (Roche Applied Science) with 1 μl cDNA per 20 μl, 4 mM MgCl_2_, 0.4 μM each primer, in a LightCycler 480 (Roche Applied Science) for 40 cycles: 10 s at 95°C, 5 s at 60°C, 10 s at 72°C. The threshold cycle (Ct) value, corresponding to the PCR cycle number at which fluorescence was detected above threshold, was calculated from Lightcycler 480 software version 1.3 (Roche Applied Science) by using the second derivative maximum method. Relative mRNA values were calculated with RealQuant Software (Roche Applied Science) by using HPRT as the reference gene.

### Immunocytochemical quantification of STAT3 and phospho-STAT3 on neurospheres

One day before stimulation with leptin (i.e., at 13 DIV), one-half of the culture medium was changed to MEM with 2% B27 supplement. To reduce the basal level of STAT3 phosphorylation, cultures were incubated for 30 min in TTX (1 μM). The cultures were then stimulated with leptin (6.2 nM) for 5, 10, or 20 min. After stimulation, all culture wells were fixed with buffered 4% formaldehyde at 4°C and rinsed several times. Coverslips were then pre-incubated in PBS-Triton X-100 (0.1%)-goat serum (5%) for 1 h at room temperature and incubated overnight with rabbit antiphospho-STAT3 (pSTAT3) or rabbit anti-STAT3 (Cell Signalling Technology Inc. Danvers, MA, USA) and with mouse anti-MAP2 (Sigma) primary antibodies. Alexa 488-conjugated goat anti-rabbit IgG (FluoProbes, France) and Cy3-conjugated goat anti-mouse IgG (Jackson ImmunoResearch Laboratories, Inc., PA, USA) were used as secondary antibodies. All procedures were performed in phosphate-free solution containing 140 mM NaCl, 5 mM KCl, and 10 mM HEPES-Na, pH 7.4.

Images were acquired with a LSM 510 laser-scanning confocal microscope (Zeiss, Germany). The acquisition of A488 (pSTAT3 or STAT3) and then Cy3 (MAP2) was sequential to avoid overlap of excitation and emission of fluorescence. The optical sections were digitized (1024 × 1024 pixels) and processed using Image J software. Ten randomly chosen optical fields were analyzed from each experiment (3–15 neurons per field). For analysis of the intensity of pSTAT3 or STAT3 staining in neuronal cells, we first created a binary mask from MAP2-positive cells and then analyzed pSTAT3/STAT3 intensity in regions overlapping with the binary mask. This procedure allowed avoiding detection of pSTAT3 in non-neuronal cells. All acquisitions and analysis were done blind. Acquisition parameters were same for every set of experiments. STAT3 immunostaining was performed in parallel cultures treated in the same condition as for pSTAT3 experiments. The pSTAT3 to STAT3 intensity ratio was expressed as means value ratio of the pSTAT3 staining intensity vs. the STAT3 staining intensity in cultures run in parallel.

### Western blotting

Protein extraction from 2 to 10 rats and Western blotting were performed as described previously (Fombonne et al., 2007). In brief, soluble neurosphere extracts were adjusted at 30 μg protein/sample after protein quantification with the BCA Protein Assay Kit (Pierce, Rockford), resolved through 4–20% Tris-Glycine gels (Invitrogen) and transferred to nitrocellulose membranes. Membranes were incubated overnight at 4°C with a relevant primary antibody: rabbit monoclonal anti-cyclin D1 (NeoMarker, 1:500), rabbit polyclonal anti-phospho(Thr202/Tyr204)p42/p44 ERK, anti-phospho(Thr308)AKT, or anti-phospho(Tyr705)-STAT3 (Cell Signaling Technology, 1: 1000), then 2 h at room temperature with horseradish peroxidase (HRP)-conjugated goat anti-rabbit IgG (1:3000) (Santa Cruz Biotechnology, Paso Robles, CA, USA). After rinsing in PBST-tween and incubation for 1–5 min in ECL-Plus reagent (Perkin-Elmer, Waltham, MA, USA), nitrocellulose membranes were exposed to Hyper Performance Chemiluminescence film. Subsequent to cyclin D1 detection, the membranes were re-incubated with rabbit polyclonal anti-β-actin antibody (Sigma, 1:10,000) according to the same experimental procedure to serve as a loading control. After detection of each phosphorylated protein, the bound antibodies were stripped off and the membranes were reblotted with either rabbit anti-p42/p44 ERK, anti-AKT, or anti-STAT3 polyclonal antibodies (Cell Signaling Technology, 1:1000). Immunolabeled bands were quantified using the public domain NIH Image program (National Institutes of Health, Bethesda, MD, USA). The relative amount of cyclin D1 protein was measured and expressed as the ratio over β-actin expression whereas the amounts of phosphorylated proteins were determined as a ratio over relevant total protein expression.

### Cyclin D1-specific shRNA design and transfections

The sequence of cyclin-specific shRNA of the cyclin D1 (CCND1) gene was designed by using siRNA Target Designer (www.promega.com/siRNADesigner/program/default.asp) soft-ware. Among designed sequences, the one corresponding to the coding region (233–255) was chosen for single-strand cDNA synthesis. The chosen forward sequence was: 5′TTTGA CCTGCGCGCCCTCCGTTTCTTTCAAGAGAAGAAACGGAGGGCGCGCAGGTCTTTTT 3′ and the chosen reverse sequence was 5′CTAGAAAAAGACCTGCGCGCCCTCCGTTTCTTCTCTTG AAAGAAACGGAGGGCGCGCAGGT 3′. After annealing of synthesized single strand cDNAs, the resulting shRNA was ligated into mU6pro vector (Promega) and resulting plasmid was amplified as previously described (Efthimiadi et al., 2012). The primers used to identify mU6pro vector containing the chosen shRNA sequence were: F1F (CATTCAGGCTGCGCAACTGTTG) and M13R2 (CACAGGAAACAGCTATGACCAT) giving rise to an amplified fragment of 810 bp. After the PCR verification, plasmidic DNA was purified by using plasmid mini-prep kit (Invitrogen), the presence of cDNA sequence corresponding to the chosen shRNA was checked by sequencing (GATC Biotech, Marseille) and the relevant cDNA was amplified by using plasmid maxi-prep (Invitrogen).

Transfection experiments were performed by using tertiary neurosphere cultures with plasmidic cDNAs corresponding to pEGFP (BD Biosciences) and generated mU6pro-cyclinD1 shRNA and NeuroMag® magnetofection kit (OZ Biosciences) according to the manufacturer's instructions. Briefly, 0.3 μg of cDNAs corresponding to pEGFP were transfected alone to serve as a transfection contol or in combination with 0.7 μg of cDNAs corresponding to mU6pro-cyclinD1 shRNA. After 24 h-period of transfection, the cell cultures were treated or not with leptin (6.2 nM). At the end of the treatments, the neurospheres were fixed with 4% paraformaldehyde for immunocytochemistry.

### Statistical analysis

Immunoblotting intensities and enumeration data obtained from cytochemical studies were compared between experimental groups with One-Way ANOVA followed by Newman-Keuls (for cellular assays) or Fisher's PLSD (for Western blots) post-tests for multiple comparisons. Unpaired Student's *t*-test (Prism software, Graph-Pad, San Diego, CA, USA) was used in experiments where only two sets of values were compared.

## Results

### SVZ-derived neurospheres respond to exogenous leptin by decreased expansion and express endogenous ObRb receptor transcripts

Microdissected SVZ tissues yielded 90,000 dissociated cells per rat. The majority of initially seeded cells died in agreement with their well-known low survival rate in the standard neurosphere culture conditions (Louis et al., 2013). Surviving cells proliferated, giving rise to growing spherical masses of undifferentiated cells. Plating at 10,000 cells per ml, when cultured 10 days *in vitro* (DIV) in the presence of 20 ng/ml of mitogens (EGF and bFGF), yielded 1700–1900 neurospheres per rat SVZ pair. The primary cultures of SVZ neurospheres were passaged at 10–11 DIV, i.e., before sphere diameter exceeded 100–120 μm in size. Passaging cells after dissociation of these neurospheres gave rise to morphologically similar neurospheres up to five successive generations. Upon differentiation by retrieval of growth factors after 10 DIV, the three neural lineages (neurons, astrocytes, oligodendrocytes) could be identified by immunocytochemistry of phenotypic markers (βIII-tubulin, GFAP, O4 respectively, data not shown) as already reported in our previous study (Charrier et al., 2006). This indicated that the cells comprised in the neurospheres fulfill the criteria for *bone fida* adult neural stem cells (Louis et al., 2013). Besides, during these preliminary experiments and the set-up of optimal culture conditions, we observed that reducing EGF concentration in the culture medium to 8 ng/ml still allowed neurosphere yield of 70–75% the values obtained with 20 ng/mL EGF at 10 DIV from adult SVZ tissues.

**Figure 1:**
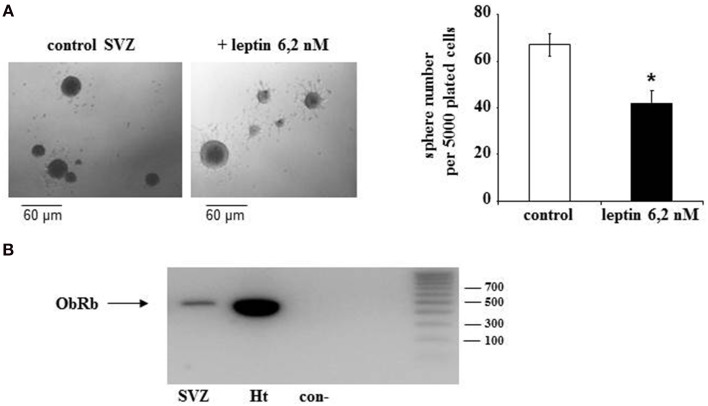
**Morphological effect of leptin and expression of its receptor (ObR) in adult rat SVZ neurospheres**. **(A)** Morphology of primary neurospheres. Neurospheres were obtained from microdissected adult rat SVZ at 10 DIV. The neurospheres were cultured in the presence of EGF and bFGF (8 ng/mL each), in the absence (control) or in the presence of leptin (6.2 nM). Arrows point to typical examples of neurospheres. Neurosphere counts on histograms are given as means ± s.e.m. of three independent experiments. ^*^significantly different from control at *p* < 0.05. **(B)** Endogenous expression of leptin receptor mRNA by SVZ-derived neurospheres. RT-PCR and gel electrophoresis detection of ObRb transcripts in mRNA extracts of SVZ neurospheres (lane 1), whole rat hypothalamus (lane 2), vs. internal control in the absence of cDNA template (con-, lane 3) and commercial DNA standard mix (lane 4). Base pair standard values are indicated on the right.

In all further experiments leptin effects on neurosphere expansion were therefore systematically assessed in the presence of 8 ng/mL EGF. Treatment with 6.2 nM leptin (Bariohay et al., 2005) strongly inhibited neurosphere growth in adult SVZ cultures as compared to controls carried in the presence of EGF but in the absence of leptin (Figure 1A). At 10 DIV, the number of neurospheres in leptin-treated SVZ cultures fell by 35% (Figure 1A).

The first step in addressing the molecular mechanisms behind leptin actions on SVZ neurospheres was to ascertain that in our experimental conditions the neurospheres express the specific leptin receptor (ObRb). As in hypothalamus, which served as a positive control, RT-PCR performed on the whole RNAs extracts obtained from SVZ-derived neurospheres yielded a specific transcript of the expected 438-bp size corresponding to ObRb leptin receptor (Figure 1B).

### Leptin recruits two ObRb post-receptor pathways in SVZ neurospheres

**Figure 2:**
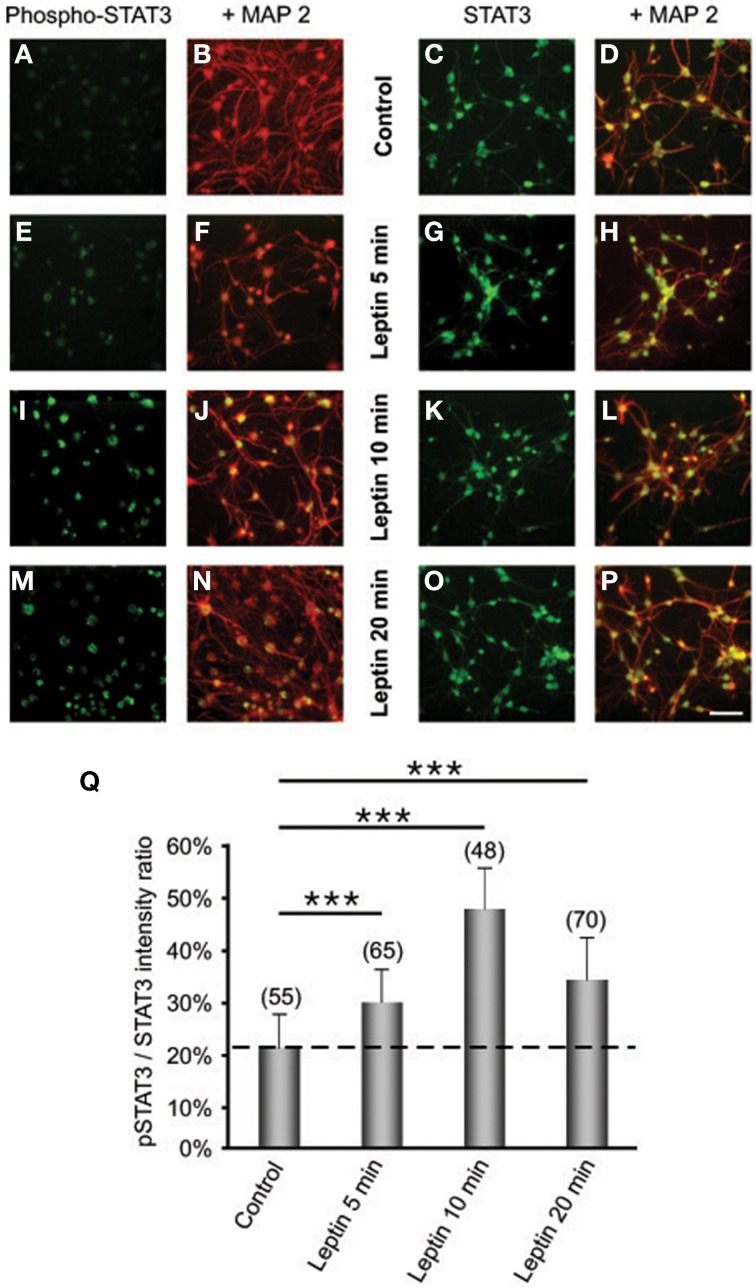
**Leptin triggers STAT3 pathway activation in SVZ-derived neurospheres**. **(A–P)** Immunocytochemical assay of leptin effects on STAT3 **(C,D,G,H,K,L,O,P)** and its active phosphorylated form (Phospho-STAT3; **A,B,E,F,I,J,M,N**) in MAP2-labeled neuronal cell subpopulation of in SVZ neurospheres. SVZ neurospheres were assayed in the absence (Control) or in the presence of 6.2 M leptin for the indicated time periods. ^***^significantly different from control at *p* < 0.001. **(Q)** Proportions (%) of activated Phospho-STAT3 immunoreactive among total STAT3 cells; the numbers in brackets indicate the number of STAT-3 cells quantified for each duration of leptin exposure.

To explore whether detected ObRb mRNA expression corresponds to the presence of functional leptin receptors, we first studied if leptin could alter STAT3 expression and phosphorylation in SVZ neurospheres by double immunocytochemistry and confocal microscopy. In both control and leptin-treated neurospheres, unphosphorylated (inactive) STAT3 was expressed only in MAP2-immunoreactive, i.e., neuronal cells; reciprocally all neuronal cells displayed STAT3 immunoreactivity (Figures 2 C,D,G,H,K,L,O,P). In the absence of leptin, the phosphorylated form of STAT3 (pSTAT3) was almost undetectable in neurosphere cultures (Figures 2A,B). However, leptin triggered rapid and transient STAT3 phosphorylation which peaked 10 min after its addition (Figures 2 E,I,M,Q) and was restricted to MAP2-labeled neuronal cells (Figures 2 E,F,I,J,M,N).

We also assessed the putative activation of the AKT-phosphoinositide-3 (PI3) pathway by leptin in SVZ neurospheres, since this pathway is generally activated in leptin targets relevant to energy metabolism regulation (Coppari and Bjørbæk, 2012). No activation-specific phosphorylation of amino-acid residues (Thr^308^) in AKT was detectable (data not shown), thus indicating that this pathway is not involved in signaling leptin actions on SVZ neurospheres.

**Figure 3:**
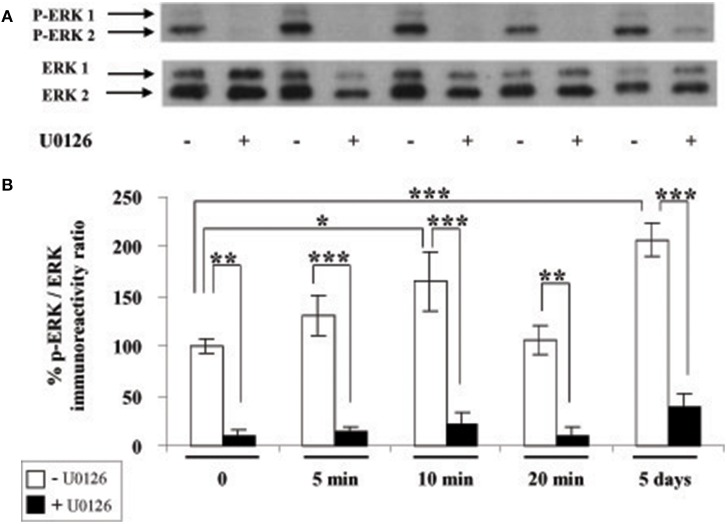
**Leptin triggers ERK pathway activation in SVZ-derived neurospheres**. **(A)** Western-blot of phosphorylated ERK 1, 2 (P-ERK1, P-ERK2) and of total ERK1 and ERK2 performed on soluble protein extracts obtained from SVZ neurospheres that were plated and cultured in the presence of 6.2 nM leptin without (−) or with (+) 10 μM U0126 for the indicated time periods. **(B)** Densitometric quantification of chemiluminescence-revealed immunoreactivities is depicted on the histogram as the ratio of phospho-ERK vs. total ERK labeling. ^*^, ^**^, ^***^significantly different from control at *p* < 0.05, *p* < 0.01, and *p* < 0.001, respectively.

We next investigated whether leptin actions also involve the ERK pathway. We found that addition of leptin to SVZ cultures triggers an increased phosphorylation of critical threonine/tyrosine residues (Thr^202^/Tyr^204^) in both ERK1 and ERK2 (p44 and p42, respectively; Figure 3A). This increase in P-ERK pointing to ERK activation could be detected 5 min after leptin addition and was maintained over control levels up to 5 DIV (Figure 3B). Interestingly, leptin-mediated activation of ERK was the most pronounced after 5-days treatment (Figure 3). The specificity of leptin-mediated ERK activation was assessed by using U0126, an inhibitor of the upstream kinase (MEK1) that selectively activates ERK1/2. At the concentration used (10 μM), U0126 markedly inhibited leptin-induced ERK activation at all time points studied, from 5 min to 5 days (Figure 3).

The results from this set of experiments therefore indicated that endogenously expressed ObRb receptor transcripts correspond to the leptin receptors that are functionally coupled to STAT3 and ERK signaling pathways. Leptin receptor coupling to STAT3 and ERK transduction pathways appears selective since another major leptin-activated signaling pathway, AKT-PI3, is not involved in the leptin actions on adult rat neurospheres.

### Leptin increases cyclin D1 expression via ERK1/2 in cultured SVZ neurospheres

**Figure 4:**
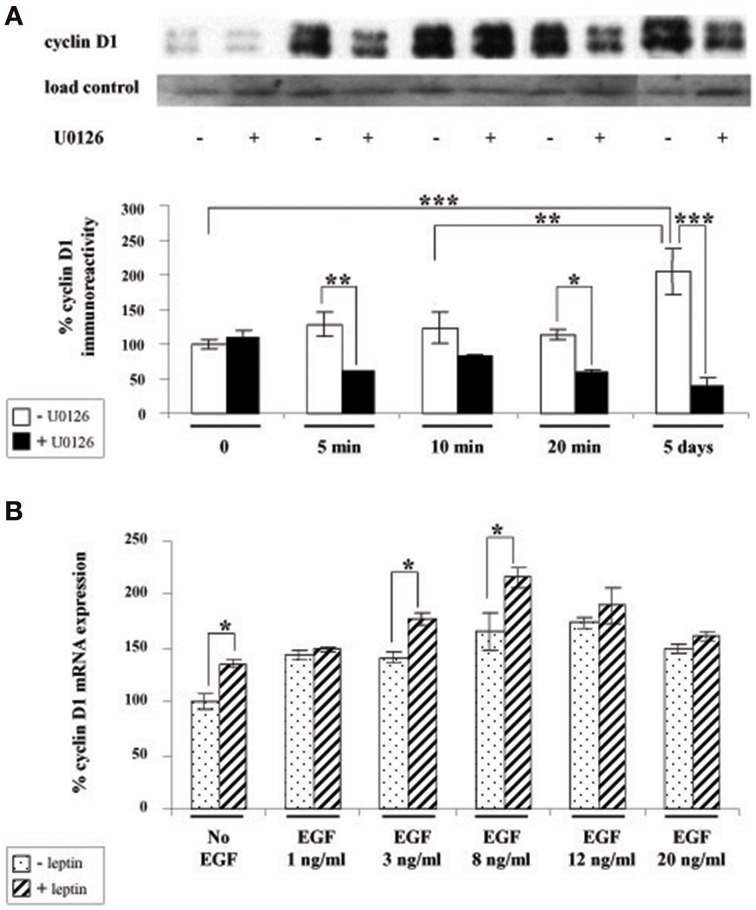
**Leptin triggers cyclin D1 induction via ERK activation in SVZ-derived neurospheres**. **(A)** Western-blot of cyclin D1 on protein extracts of SVZ neurospheres cultured in the presence of 6.2 nM leptin without (−) or with (+) 10 μM U0126 for the indicated time periods. Load control was provided by β-actin western-blot after stripping of the cyclin D1-labeled membranes. The intensity of cyclin D1 expression was normalized over respective β-actin labeling used as a loading control and expressed as % of the time 0 expression. **(B)** qRT-PCR of cyclin D1 performed on mRNA extracts of SVZ neurospheres that were plated and cultured for 5 DIV with or without 6.2 nM leptin, in the presence of increasing EGF concentrations. Data are expressed as % of cyclin D1 expression signal in the absence of leptin and EGF. ^*^, ^**^, ^***^significantly different at *p* < 0.05, *p* < 0.01, and *p* < 0.001, respectively.

Given that cyclin D1 is one of the major regulators of cell division and growth (Sherr and Roberts, 2004) and the reported involvement of ERK pathway in the control of cyclin D1 expression (Lefloch et al., 2009), we sought to determine whether leptin might alter cyclin D1 expression in SVZ neurospheres. Indeed, since leptin inhibited the growth of neurospheres in our *in vitro* assay, we hypothesized that leptin may also inhibit cyclin D1 expression via ERK activation. Unexpectedly, as indicated by cyclin D1 western blot (Figure 4A), leptin clearly did not trigger a decrease in cyclin D1 expression. Moreover, after 5 DIV, the cyclin D1 expression was increased almost two-fold over basal expression (Figure 4A). The ERK1/2 inhibitor U0126 could reduce leptin-triggered induction of cyclin D1 protein to at least one-half of that seen in the absence of the inhibitor (Figure 4A) thus pointing to a specific involvement of ERK1/2 in leptin's action on cyclin D1 expression. Leptin-dependent induction of cyclin D1 was confirmed at the transcriptomal level by qRT-PCR at the time point (i.e., 5 DIV) where the maximal cyclin D1 protein expression was observed (Figure 4B). Indeed, after 5 DIV, leptin consistently increased the expression of cyclin D1 transcripts by itself (Figure 4B: no EGF condition). This effect was comparable to the cyclin D1 mRNA induction by the mitogen EGF (in the absence of leptin), used here as a positive control.

The above combined biochemical and pharmacological approaches therefore show that leptin increases cyclin D1 expression. This leptin effect is synergistic with the EGF-mediated cyclin D1 induction thus suggesting that leptin and EGF mediate the increase in cyclin D1 via different mechanisms.

### Leptin triggers withdrawal from the cell cycle and cell death in SVZ neurospheres

**Figure 5:**
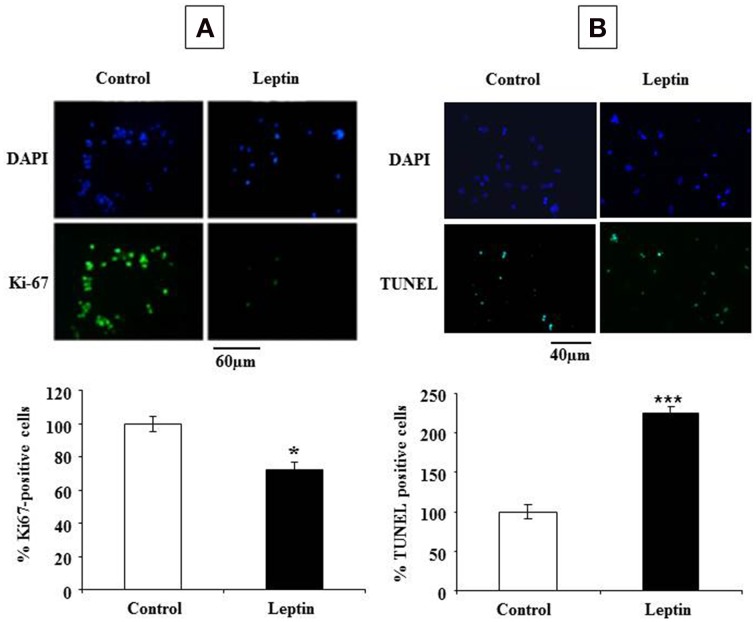
**Leptin inhibits proliferation and triggers cell death of SVZ neurospheres**. **(A)** Photomicrographs of DAPI staining (top row) and Ki-67 green immunocytofluorescence (lower row) in control (no leptin) and 6.2 nM leptin-treated neurosphere cultures at 5 DIV. **(B)** Photomicrographs of DAPI staining (top row) and TUNEL green cytofluorescent staining (lower row) in control (no leptin) and 6.2 nM leptin-treated neurosphere cultures at 5 DIV. Histograms represent the proportions of Ki-67 **(A)** or TUNEL **(B)** positive nuclei expressed as % of the total cell number (as determined by DAPI staining) by taking as a reference 100% the values obtained for the control condition. Data are expressed as mean percent of control ± SEM. Statistical significancies at ^*^p < 0.05 or ^***^p < 0.001.

The observed leptin-mediated increase of cyclin D1 (Figure 4) appeared paradoxical in the light of our findings concerning the inhibition of neurosphere neural cell proliferation by leptin (Figure 1). To understand the mechanism behind such paradoxical effect of leptin, we checked directly leptin effects on the cell cycle by immunocytochemical analysis of Ki-67 labeling. This marker is expressed by cycling cells in any phase of the cell cycle but not by quiescent cells (Scholzen and Gerdes, 2000). In the absence of leptin, the majority of cells (about 60% of the total cell population as identified by DAPI-staining of their nuclei) were in the cell division cycle (Figure 5A). Addition of leptin resulted in a significant decrease in the proportion of the Ki-67-positive cells as compared to control cultures (Figure 5A).

The inhibitory actions of leptin on neurosphere growth are therefore associated with the inhibition of cell division in the presence of leptin but not with an expected decrease in cyclin D1 expression. Because in some paradigms of neuronal death, induction of cyclin D1 precedes the cell death (Di Giovanni et al., 2005; Krantic et al., 2005), we asked whether leptin could trigger cell death in our neurosphere assays. Assessment of apoptosis by TUNEL staining indicated that, indeed, leptin treatment yielded a two-fold increase of the number of TUNEL-positive cells (Figure 5B).

### Cyclin D1 expression in tunel-positive neurosphere cells after leptin treatment

**Figure 6:**
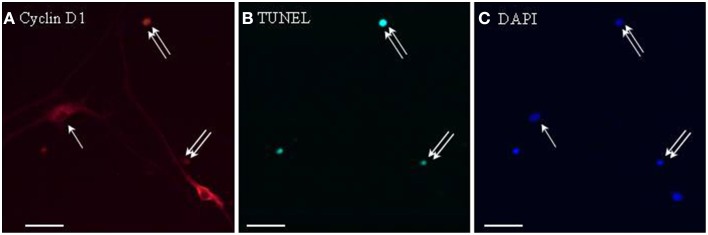
**Cyclin D1 immunoreactivity colocalizes with TUNEL-positive cells in leptin-treated neurospheres**. A series of identical culture wells of leptin-treated secondary neurospheres from adult rat SVZ were processed in parallel for either **(A)** cyclin D1 immunocytochemistry (revealed in red), **(B)** TUNEL (revealed in green), **(C)** DAPI staining (revealed in blue). Single and double arrows point respectively, to TUNEL−/cyclinD1+ and TUNEL+/cyclinD1+ representative cells. Note the occurrence of TUNEL-negative cyclin-D1-immunoreactive cells, and the differential morphologies of the two subpopulations of labeled cells (see Results text for details).

To address whether cyclin D1 induction could be involved in leptin-stimulated apoptosis, TUNEL staining was combined with cyclin D1 immunocytochemistry on proliferating SVZ neurosphere cultures. Cyclin D1 immunoreactivity was much more abundant in leptin-treated neurospheres than in control cultures. In leptin-treated neurospheres, cyclin D1-immunoreactive cells splitted into two strikingly different subpopulations: small cells with cyclin D1 restriction in the cell nucleus, and large neuron-like cells expressing cyclin D1 throughout cytoplasm but not in nuclei (Figure 6). TUNEL staining was extensively and exclusively colocalized with nuclear cyclin D1, and never associated with cytoplasmic cyclin D1-containing cells (Figure 6).

### Silencing cyclin D1 expression by specific shRNA prevents leptin-induced decrease of the neural stem cell number

**Figure 7:**
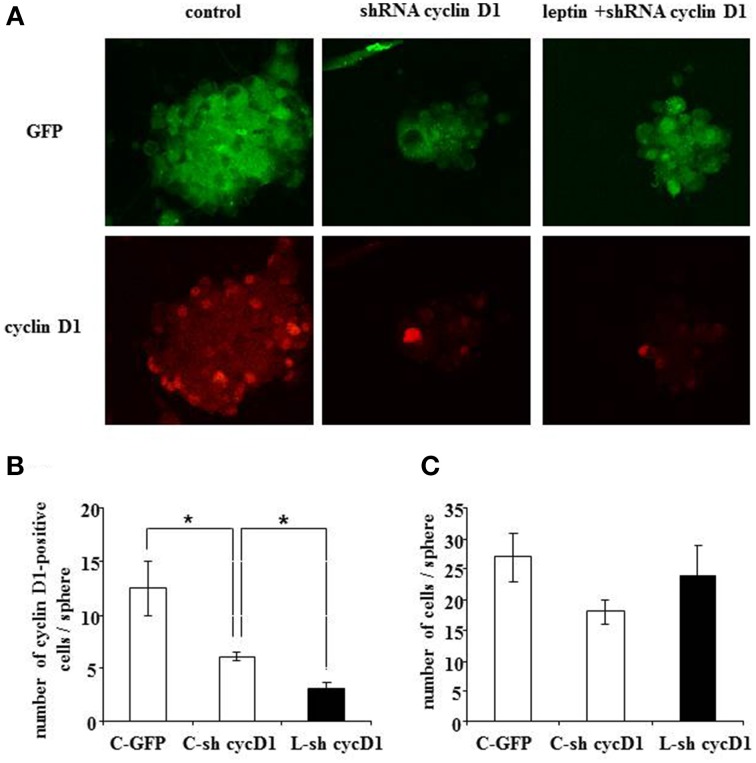
**Leptin-triggered cell death is inhibited by shRNA-silencing of cyclin D1 expression in SVZ neurospheres**. **(A)** GFP fluorescence (top row) and cyclin D1 immunocytofluorescence (bottom row) in neurospheres with single GFP transfection in the absence of leptin (first column from left, “control”) or with double GFP- and shRNA cyclinD1- transfection in the absence (second column) or in the presence (third column) of 6.2 nM leptin. **(B)** Quantification of cyclinD1-immunoreactive cells per neurosphere in the three experimental conditions. **(C)** Enumeration of cells per neurosphere in the three cultures run in parallel. Data are expressed as mean ± SEM of three independent cultures. ^*^ significantly different from control at *p* < 0.05.

To assess a causal relationship between leptin-mediated cyclin D1 expression and cell death, we performed RNA silencing experiments with a cyclin D1-specific shRNA. Knock-down of cyclin D1 by specific shRNA resulted in a decrease of the number of cyclin D1-positive cells per neurosphere (Figures 7A,B), thus confirming the functionally efficient knock-down of cyclin D1 expression.

Moreover, when cyclin D1-specific shRNA-transfected neurospheres were treated with leptin (6.2 nM), a decrease in the number of cyclin D1-labeled cells due to the transfection could not be over-come by leptin-mediated increase in cyclin D1 expression (Figure 7A: cyclin D1 row, and Figure 7B for quantification) seen in previous experiments (Figure 4). Interestingly, such decrease in the number of cyclin D1-labeled cells precluded the leptin triggered-decrease in the number of cells per neurosphere (Figure 7A: GFP row, and Figure 7C for quantification).

Taken together, these data show that functional knock-down of cyclin D1 expression is sufficient to rescue leptin-mediated decrease in the cell number per neurosphere.

### Phenotypic identification of apoptotic cells in leptin-treated neurospheres

**Figure 8:**
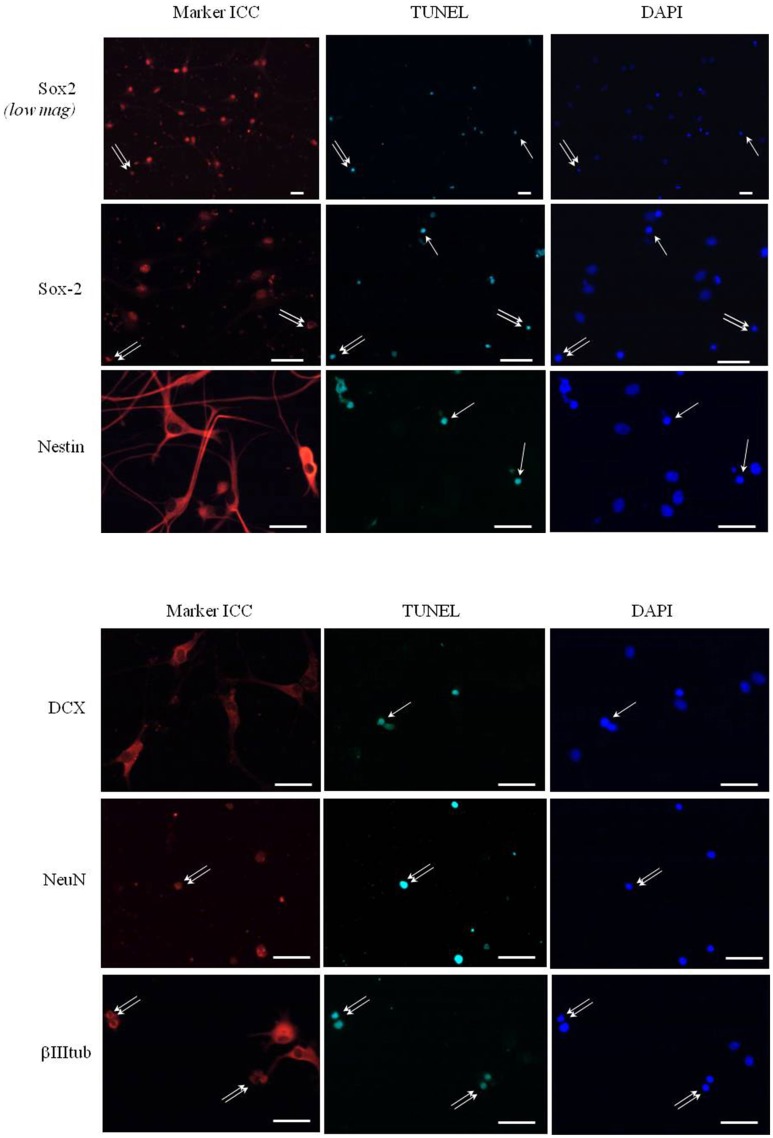

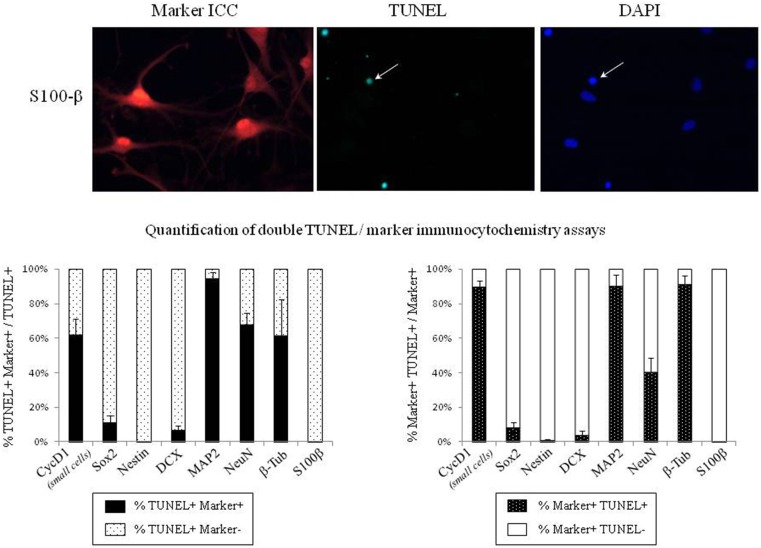
**Phenotypic identification of leptin-induced apoptotic cells in SVZ neurospheres**. Secondary neurospheres from adult rat SVZ were grown on poly-lysine-coated coverslips in the presence of EGF, bFGF (8 ng/mL each) and leptin (6.2 nM), fixed after 5 DIV, processed for TUNEL (kit Roche, green fluorescent detection, middle column), then for red Alexa-594 fluorescence-revealed marker immunocytochemistry (left column), and finally for DAPI staining (right column). Each row displays photomicrographs of the same representative field from one neurosphere culture that was processed for immunocytochemistry of the marker indicated on left and observed with respective fluorescence-activating lights for each of the three labelings indicated on column top. Scale bars: 30 μm. Single and double arrows point respectively to TUNEL+/marker− and TUNEL+/marker+ representative cells. Quantitative analyses of each marker/TUNEL combination are presented on the two histograms at the bottom; each value is mean ± SEM of three cultures.

In order to identify the cell types that are committed to apoptosis in response to leptin among the neuropoietic lineage, TUNEL was combined with immunocytochemistry of each among eight phenotypic markers on neurosphere cultures from adult rat SVZ (Figure 8). Proportions of marker-immunoreactivity among TUNEL-positive cells and of TUNEL positivity among each phenotypic subpopulation of SVZ neurosphere cells were quantified on large numbers of cells in three distinct cultures at least (above 100 in each case) and respectively plotted in histograms of Figure 8. The neural stem cell marker Sox-2 rarely co-localized with TUNEL, although numerous TUNEL-positive and Sox-2 immunoreactive cells were detected (Figure 8, two first rows). No TUNEL colocalization was detected with the other neural stem cell marker, nestin (Figure 8). A small proportion of TUNEL colocalization was detected with the marker of immature migrating neurons doublecortin (DCX) (Figure 8). By contrast, TUNEL staining extensively colocalized with three markers of differentiated neurons: the microtubule-associated protein-2 (MAP2), the neuronal nuclear antigen NeuN and the axonal protein β-III-tubulin (Figure 8). No TUNEL colocalization was detected with the astrocyte marker S100β (Figure 8).

## Discussion

The present study provides the first report on leptin-mediated inhibition of neural stem cell expansion in neurospheres derived from the adult rat neurogenic niche, SVZ. These leptin actions are mediated, at least in part, by apoptosis involving cyclin D1 induction as a prelude to cell death. The characterized novel actions of leptin on adult brain neural stem cells involve receptors and overlapping post-receptor mechanisms also relevant for its classical effects on energy metabolism.

Our neural stem cell culture conditions were validated by the fact that in control conditions, the primary cultures from adult rat SVZ yielded similar growth kinetics as those previously reported by our group and others (Charrier et al., 2006; Louis et al., 2013). *In vitro* inhibition of neurosphere expansion is due to a decrease in the number of stem cells (Louis et al., 2013), which in turn results from either neural stem cell death or entry into quiescence (Daynac et al., 2013). In our experiments, the inhibitory effect of leptin on neural stem cell expansion could be revealed *in vitro* by lowering the EGF concentration from standard 20 ng/mL (Louis et al., 2013; Charrier et al., 2006) to 8 ng/mL, in agreement with previous studies (Palma et al., 2004; Louis et al., 2013).

The novel actions of leptin characterized in the present study involve the receptor molecule, ObRb, shared by all known leptin target cells. Indeed, ObRb transcripts were amplified from SVZ neurosphere RNAs by using primers designated for amplification of the conserved cDNA sequences. Although the performed RT-PCR assay does not allow for the accurate quantification, ObRb expression level in SVZ neurospheres appears lower than in hypothalamus, which is one of the major leptin targets in adult mammals organism (Elmquist et al., 1998; Villanueva and Myers, 2008; Myers et al., 2009; Gautron and Elmquist, 2011; Coppari and Bjørbæk, 2012; Park and Ahima, 2014). The functionality of ObRb in SVZ neurospheres is attested by leptin activation of the specific transduction pathways: STAT3 and ERK1/2. In particular, phospho-STAT3 immunoreactivity is established as a universal index of functional recruitment of leptin receptor in target cells. Indeed, among the five intracellular pathways that can be recruited by leptin reception in its numerous target cell types, the Jak2/STAT3 is the only one that is systematically activated by leptin (Villanueva and Myers, 2008; Coppari and Bjørbæk, 2012; Rasmussen et al., 2014). Interestingly, STAT3 phosphorylation-dependent activation was exclusively detected in MAP2-immunoreactive cells. As a corollary, the STAT3 transduction pathway may be involved in leptin-mediated induction of differentiation via commitment of the neural stem cells to neural progenitors. Differentiation-related actions of leptin were out of the scope of this study. It is nevertheless likely that commitment of neural stem cells to differentiation toward a neural lineage may be preceded by the observed withdrawal of neural stem cells from the cell cycle, as shown by our Ki67 labeling experiments. Ki67 is a nuclear protein which is exclusively expressed by dividing cells (Scholzen and Gerdes, 2000). The observed decrease in Ki67 labeling in the presence of leptin therefore points to the inhibitory role of leptin in the control of SVZ neural stem cell expansion. This novel action of leptin on neurospheres adds to the well-known complexity of leptin-mediated control of cell division pointing to both inhibitory and stimulatory effects of leptin (reviewed in Garofalo and Surmacz, 2006; King et al., 2013). For instance, anti-proliferative actions of leptin combined with leptin-mediated induction of cell death, have been documented in immune, epithelial, endothelial, adipose cells, and some tumors (Garofalo and Surmacz, 2006; Tang, 2008). In line with these findings, the inhibitory effect of leptin on adult SVZ neural stem cell expansion shown here was also associated to apoptosis induction. Moreover, apoptosis was preceded by ERK-mediated cyclin D1 induction. In agreement, ERK has been previously shown to be involved in the control of cyclin D1 expression (Meloche and Pouyssegur, 2007). The specificity of ERK mediation was further attested by the capacity of the ERK kinase MEK1 inhibitor (U0126) to prevent cyclin D1 induction and cell death upon leptin addition to SVZ neurospheres. At the cellular level, cyclin D1 expression by the apoptotic cells was directly demonstrated by colocalization of cyclin D1 immunoreactivity and TUNEL staining in numerous nuclei of leptin-treated neurosphere cultures from adult rat SVZ. Furthermore, this experiment revealed that a subpopulation of differentiated, TUNEL-negative, neuron-like cells displayed strong cytoplasmic accumulation of cyclin D1 protein. It suggests that leptin-dependent induction of cyclin D1 might trigger either apoptosis or differentiation of neurosphere cells.

The specificity of cyclin D1 involvement in cell death induction was demonstrated by our RNA silencing experiments. Interestingly, cyclin D1 silencing in the absence of leptin resulted in withdrawal from the cell cycle as shown by decreased Ki67 expression. In these control conditions, the basal cyclin D1 expression by neurosphere cells is likely due to the presence of a low level of EGF concentration in culture medium. Indeed EGF, like other mitogens, has the capacity to trigger cyclin D1 expression and subsequent entry into the cell division cycle (Sherr and Roberts, 2004; Meloche and Pouyssegur, 2007). Therefore, in the absence of leptin, cyclin D1 likely acts in its normal capacity as a positive regulator of the cell division cycle (Sherr and Roberts, 2004). However, because it has been demonstrated that the over-expression of cyclin D1 leads to increased proliferation in immature, i.e., cycling, neurons (Oliver et al., 2003) whereas it triggers cell death in mature, i.e., differentiated, neurons (Kranenburg et al., 1996; Timsit et al., 1999), our data suggest that the present pro-apoptotic action of leptin might be associated with its concomitant pro-differentiative effects on SVZ cells. This hypothesis is supported by the following experimental evidence: (i) morphological signs of differentiation (neurite growth, cell migration), similar to those seen in in pro-differentiative culture conditions such as mitogen retrieval, were consistently observed in the presence of leptin; (ii) this leptin-dependent differentiation is associated with cyclin D1 in the cytoplasm of neurosphere cells; (iii) leptin triggers neural stem cell withdrawal from the cell cycle, as monitored by decreased Ki67 labeling, which is generally required for induction of cell differentiation (Gao and Zelenka, 1997). Relevantly, it has been demonstrated already that failure in differentiation leads to cell death in other paradigms such as in EGF-induced differentiation of somato-lactotrope precursors into PRL-secreting lactotrope cells (Fombonne et al., 2004) or in erythropoietin-induced differentiation of hematopoietic lineage (Lesault et al., 2002).

Leptin targets for commitment to apoptosis in SVZ neurospheres, i.e., in the “neuropoietic lineage,” were identified by a double TUNEL/immunocytochemical approach, as differentiated neurons predominantly. Less than 10% of TUNEL-positive cells indeed expressed Sox2 or DCX in the present culture conditions. These data are in keeping with the result of our previous time-course assay of leptin-dependent transduction effector, STAT-3 phosphorylation, which occurred exclusively in neurosphere neurons (see above). It indicates that leptin impacts late stages of neuropoiesis, even though its present action is detected in proliferating neurospheres and primarily targets the early cell division factor cyclin D1. Consistently, it is established that neuron differentiation markers are already expressed during the proliferative state of neural stem/progenitor cells in the presence of mitogens (Liard et al., 2009; Rosa et al., 2010). Cyclin D1 was recently shown in neural stem cells of fetal rodent brain, to block astroglial differentiation and to stimulate neuronal differentiation *via* STAT-3 activation (Bizen et al., 2014). This latter finding is similar to the presently characterized actions of leptin on adult rat SVZ cells, although it was characterized on neural progenitors from late fetus. Further, our cytochemical assays clearly showed that leptin-induced cyclin D1 colocalizes with TUNEL-stained apoptosis in the nuclei of small SVZ cells, whereas cyclin D1 is also extensively expressed in much larger, neuron-like, non-apoptotic cells. This dual subcellular distribution of cyclin D1 is suggestive of its alternative roles that have recently emerged and are independent of cell cycle onset (Pestell, 2013).

From a more integrated point of view, the presently characterized leptin actions on adult SVZ neural stem cells provide a putative mechanism for *in vivo* leptin-dependent physiological adaptations. Functional deficiency of leptin actions in genetic (db/db mice) (Ramos-Rodriguez et al., 2014) or physiological (long-term intermittent fasting, Manzanero et al., 2014) models leads to increased neural stem cell proliferation in adult rat SVZ. Likewise, daily exercise in adult rats has been reported to increase neurogenesis in the adult hippocampus while decreasing leptinemia (Speisman et al., 2013). In contrast, increased levels of leptin, resulting from a high-fat diet, inhibit neurogenesis in the adult mouse hippocampus without neuronal loss (Park et al., 2010). Consistently, obesity decreases the number of neurosphere-producing cells in hypothalamus-derived primary neurosphere cultures (Bless et al., 2014). The putative leptin-dependent decrease of neurogenic rate in adult olfactory bulb, as suggested by the present *in vitro* study, should alter olfactory perception since odor discrimination and memory were recently demonstrated to depend on adult SVZ-fed neurogenesis in olfactory bulb (Gheusi and Lledo, 2014). The relevance of leptin impact on SVZ neural stem cells for food intake regulation though appears complex since olfactory perception could be optimized both by net increase (Sultan et al., 2010) and decrease (Mouret et al., 2009) of olfactory bulb neurogenesis in adult rat.

In conclusion, the present data further extend the known role of leptin in the control of neural plasticity in adult mammals. More precisely, since our data show that *in vitro* leptin inhibition of adult neural stem cell expansion is associated with specific receptor-mediated induction of apoptosis, and since *in vivo* the leptin hormone is secreted in direct proportion to adiposity, the present study suggests that adiposity/obesity may be considered as a neurotoxicity paradigm. This view is further supported by the recent *in vivo* report that a high-fat leptin-inducing diet during 2 months in adult mouse and rat triggers apoposis in mature hypothalamic neurons (Moraes et al., 2009). These recent findings extend the physiological pleiotropy of leptin beyond hormonal action toward a multifaceted growth factor in adult mammals. In this light, it is interesting to stress that the major post-receptor intracellular relays of leptin (STAT3, ERK1/2, AMPK, AKT) play a role in the control of both the cell cycle in proliferating cells and metabolite/metabolic sensing in post-mitotic neurons of the brain feeding regulatory centers (Rahmouni et al., 2009; Haissaguerre et al., 2014; Rehman et al., 2014). Stem cell control by leptin may therefore provide an exciting paradigm for the study of the cross-talk between these two regulation registers (Mans and Haramis, 2014). Several questions are raised by the present results about the function and relevance of leptin and leptin receptors in the SVZ neurogenic niche, such as the type(s) of leptin-receptive cells, the impact of leptin on differentiation/migration and behavioral alteration of stem cell progenies. Answering these questions definitively requires future studies that will focus on these exciting issues.

## Funding

This work was supported by CNRS, INRA, La Ligue Contre le Cancer Grand Ouest Comités de la Vienne et des Deux Sèvres, University of Poitiers, University François Rabelais of Tours. SS, SC were recipients of French Government Doctoral Fellowships (SS: Biology of Ageing; SC: Neurosciences). LE was recipient of PACA Region/INSERM Doctoral Fellowship.

### Conflict of interest statement

The authors declare that the research was conducted in the absence of any commercial or financial relationships that could be construed as a potential conflict of interest.

## Abbreviations

aCSF: artificial cerebrospinal fluid
AKT: protein kinase B
AMPK: 5′-adenosine monophosphate-activated protein kinase
bFGF: basic fibroblast growth factor
CNTF: ciliary neurotrophic factor
DAPI: diamidino-2-phenylindole
DIV: days *in vitro*
DMEM: Dulbecco's modified Eagle's medium
EGF: epidermal growth factor
ERK: extracellular signal-regulated kinase
MAPK: mitogen-activated protein kinase
ObRb: leptin receptor
PCR: polymerase chain reaction
RNA: ribonucleic acid
shRNA: short hairpin ribonucleic acid
siRNA: silencing RNA
STAT: signal transducer and activator of transcription
SVZ: subventricular zone.

## References

[B1] Bariohay B. Lebrun B. Moyse E. Jean A. (2005). Brain-Derived Neurotrophic Factor plays a role as an anorexigenic factor in the dorsal vagal complex. Endocrinology 146, 5612–5620. 10.1210/en.2005-0419 16166223

[B2] Bauer S. Moyse E. Jourdan F. Colpaert F. Martel J. C. Marien M. (2003). Effects of the a2-adrenoreceptor antagonist dexefaroxan on neurogenesis in the olfactory bulb of the adult rat *in vivo*: selective protection against neuronal death. Neuroscience 117, 281–291. 10.1016/S0306-4522(02)00757-1 12614670

[B3] Bizen N. Inoue T. Shimizu T. Tabu K. Kagawa T. Taga T. (2014). A growth-promoting signaling component cyclin D1 in neural stem cells has antiastrogliogenic function to execute self-renewal. Stem Cells 32, 1602–1615. 10.1002/stem.1613 24302516

[B4] Bless E. P. Reddy T. Acharva K. D. Beltz B. S. Tetl M. J. (2014). Oestradiol and diet modulate energy homeostasis and hypothalamic neurogenesis in the adult female mouse. J. Neuroendocrinol. 26, 805–816. 10.1111/jne.12206 25182179 PMC4476296

[B5] Bouret S. G. Draper S. J. Simerly R. B. (2004). Trophic action of leptin on hypothalamic neurons that regulate feeding. Science 304, 108–110. 10.1126/science.1095004 15064420

[B6] Bouret S. G. (2013). Organizational actions of metabolic hormones. Front. Neuroendocrinol. 34, 18–26. 10.1016/j.yfrne.2013.01.001 23357643 PMC3654157

[B7] Braun S. M. G. Jessberger S. (2014). Adult neurogenesis: mechanisms and functional significance. Development 141, 1983–1986. 10.1242/dev.104596 24803647

[B8] Charrier C. Coronas V. Fombonne J. Roger M. Jean A. Krantic S.. (2006). Characterization of neural stem cells in the dorsal vagal complex of adult rat brainstem by *in vivo* proliferation labelling and *in vitro* neurosphere assay. Neuroscience 138, 5–16. 10.1016/j.neuroscience.2005.10.046 16338085

[B9] Cheng M. F. (2013). Hypothalamic neurogenesis in the adult brain. Front. Neuroendocrinol. 34, 167–78. 10.1016/j.yfrne.2013.05.001 23684668

[B10] Coppari R. Bjørbæk C. (2012). Leptin revisited: its mechanism of action and potential for treating diabetes. Nat. Rev. Drug Discov. 11, 692–708. 10.1038/nrd3757 22935803 PMC4019022

[B11] Daynac M. Chicheportiche A. Pineda J. R. Gauthier L. R. Boussin F. D. Mouthon M. A. (2013). Quiescent neural stem cells exit dormancy upon alteration of GABAAR signalling following radiation damage. Stem Cell Res. 11, 516–528. 10.1016/j.scr.2013.02.008 23562833

[B12] Di Giovanni S. Movsesyan V. Ahmed F. Cernak I. Schinelli S. Stoica B.. (2005). Cell cycle inhibition provides neuroprotection and reduces glial proliferation and scar formation after traumatic brain injury. Proc. Natl. Acad. Sci. U.S.A. 102, 8333–8338. 10.1073/pnas.0500989102 15923260 PMC1149422

[B13] Efthimiadi L. Farso M. Quirion R. Krantic S. (2012). Cyclin D1 induction preceding neuronal death via the excitotoxic NMDA pathway involves selective stimulation of extrasynaptic NMDA receptors and JNK pathway. Neurodegener. Dis. 10, 80–91. 10.1159/000335911 22354185

[B14] Elmquist J. K. Bjørbaek C. Ahima R. S. Flier J. S. Saper C. B. (1998). Distributions of leptin receptor mRNA isoforms in the rat brain. J. Comp. Neurol. 395, 535–547. 9619505

[B15] Fombonne J. Charrier C. Goddard I. Moyse E. Krantic S. (2007). Leptin-mediated decrease of cyclin A2 and increase of cyclin D1 expression: relevance for the control of prepubertal rat Leydig cell division and differentiation. Endocrinology 148, 2126–2137. 10.1210/en.2006-1218 17303663

[B16] Fombonne J. Reix S. Rasolonjanahary R. Danty E. Thirion S. Laforge-Anglade G.. (2004). Epidermal growth factor triggers an original, caspase-independent pituitary cell death with heterogeneous phenotype. Mol. Biol. Cell. 15, 4938–4948. 10.1091/mbc.E04-07-0601 15331766 PMC524748

[B17] Gao C. Y. Zelenka P. S. (1997). Cyclins, cyclin-dependent kinases and differentiation. Bioessays 19:307–315. 9136628 10.1002/bies.950190408

[B18] Garofalo C. Surmacz E. (2006). Leptin and cancer. J. Cell. Physiol. 207, 12–22. 10.1002/jcp.20472 16110483

[B19] Gautron L. Elmquist J. K. (2011). Sixteen years and counting: an update on leptin in energy balance. J. Clin. Invest. 121, 2087–2093. 10.1172/JCI45888 21633176 PMC3104762

[B20] Gheusi G. Lledo P. M. (2014). Adult neurogenesis in the olfactory system shapes odor memory and perception. Prog. Brain Res. 208, 157–175. 10.1016/B978-0-444-63350-7.00006-1 24767482

[B21] Grill H. J. Hayes M. R. (2012). Hindbrain neurons as an essential hub in the neuroanatomically distributed control of energy balance. Cell Metab. 16, 296–309. 10.1016/j.cmet.2012.06.015 22902836 PMC4862653

[B22] Haissaguerre M. Saucisse N. Cota D. (2014). Influence of mTOR in energy and metabolic homeostasis. Mol. Cell. Endocrinol. 397, 67–77. 10.1016/j.mce.2014.07015 25109278

[B23] Hann N. Goodman T. Nadji-Samiei A. Stratford C. M. Rice R. El Agha E.. (2013). Fgf10-expressing tanycytes add new neurons to the appetite/energy balance regulating centres of the postnatal and adult hypothalamus. J. Neurosci. 33, 6170–6180. 10.1523/JNEUROSCI.2437-12.2013 23554498 PMC3736310

[B24] King B. Jiang Y. Su X. Xu J. Xie L. Standard J.. (2013). Weight control, endocrine hormones and cancer prevention. Exp. Biol Med. 238, 502–508. 10.1177/1535370213480695 23856901

[B25] Kokoeva M. V. Yin H. Flier J. S. (2005). Neurogenesis in the hypothalamus of adult mice: potential role in energy balance. Science 310, 679–683. 10.1126/science.1115360 16254185

[B26] Kranenburg O. van der Eb A. J. Zantema A. (1996). Cyclin D1 is an essential mediator of apoptotic neuronal cell death. EMBO J. 15, 46–54. 8598205 PMC449916

[B27] Krantic S. Mechawar N. Reix S. Quirion R. (2005). Molecular basis of programmed cell death involved in neurodegeneration. Trends Neurosci. 28, 670–676. 10.1016/j.tins.2005.09.011 16216345

[B28] Lee D. A. Bedont J. L. Pak T. Wang H. Song J. Miranda-Angulo A.. (2012). Tanycytes of the hypothalamic median eminence form a diet-responsive neurogenic niche. Nat. Neurosci. 15, 700–702. 10.1038/nn.3079 22446882 PMC3380241

[B29] Lefloch R. Pouysségur J. Lenormand P. (2009). Total ERK1/2 activity regulates cell proliferation. Cell Cycle 8, 705–711. 10.4161/cc.8.5.7734 19242111

[B30] Lesault I. Quang C. T. Frampton J. Ghysdael J. (2002). Direct regulation of BCl-2 by FLI-1 is involved in the survival of FLI-1-transformed erythroblasts. EMBO J. 21, 694–703. 10.1093/emboj/21.4.694 11847117 PMC125347

[B31] Liard O. Segura S. Pascual A. Gaudreau P. Fusai T. Moyse E. (2009). *In vitro* isolation of neural precursor cells from the adult pig subventricular zone. J. Neurosci. Methods 182, 172–179. 10.1016/j.jneumeth.2009.06.008 19524610

[B32] Louis S. A. Mak C. K. H. Reynolds B. A. (2013). Methods to culture, differentiate, and characterize neural stem cells from the adult and embryonic mouse central nervous system. Meth. Mol. Biol. 946, 479–506. 10.1007/978-1-62703-128-8_30 23179851

[B33] Mans L. D. Haramis A. P. (2014). Burn to cycle: energetics of cell-cycle control and stem cell maintenance. Front. Biosci. 19, 1003–1014. 10.2741/4263 24896332

[B34] Manzanero S. Erion J. R. Santro T. Steyn F. J. Chen C. Arumugam T. V.. (2014). Intermittent fasting attenuates increases in neurogenesis after ischaemia and reperfusion and improves recovery. J. Cereb. Blood Flow Metab. 34, 897–905. 10.1038/jcbfm.2014.36 24549184 PMC4013772

[B35] McNay D. E. G. Briançon N. Kokoeva M. V. Maratos-Flier E. Flier J. S. (2012). Remodeling of the arcuate nucleus energy-balance circuit is inhibited in obese mice. J. Clin. Invest. 122, 142–152. 10.1172/JCI43134 22201680 PMC3248278

[B36] Meloche S. Pouyssegur J. (2007). The ERK ½mitogen-activated protein kinase pathway as a master regulator of the G1- to S-phase transition. Oncogene 26, 3227–3239. 10.1038/sj.onc.1210414 17496918

[B37] Moraes J. C. Coope A. Morari J. Cintra D. E. Roman E. A. Pauli J. R.. (2009). High-fat diet induces apoptosis of hypothalamic neurons. PLoS ONE 4:e5045. 10.1371/journal.pone.0005045 19340313 PMC2661137

[B38] Morton G. J. Meak T. H. Schwartz M. W. (2014). Central nervous system control of food intake and body weight. Nat. Rev. Neurosci. 15, 367–378. 10.1038/nrn3745 24840801 PMC4076116

[B39] Mouret A. Lepousez G. Gras J. Gabellec M. M. Lledo P. M. (2009). Turnover of newborn olfactory bulb neurons optimizes olfaction. J. Neurosci. 29, 12302–12314. 10.1523/JNEUROSCI.3383-09.2009 19793989 PMC6666148

[B40] Myers M. G. Jr. Münzberg H. Lehninger G. M. Leshan R. L. (2009). The geometry of leptin action in the brain: more complicated than a simple ARC. Cell Metab. 9, 117–123. 10.1016/j.cmet.2008.12.001 19187770 PMC2648854

[B41] National Research Council N. R. C. (1996). Guide for Care and use of Laboratory Animals, 8th Edn. Washington, DC: The National Academies Press.

[B42] Oliver T. G. Grasfeder L. L. Carroll A. L. Kaiser C. Gillingham C. L. Lin S. M.. (2003). Transcriptional profile of the Sonic hedgehog response: a critical role for N-myc in proliferation of neuronal precursors. Proc. Natl. Acad. Sci. U.S.A. 100, 7331–7336. 10.1073/pnas.0832317100 12777630 PMC165875

[B43] Oswal A. Yeo G. (2010). Leptin and the control of body weight: a review of its diverse central targets, signaling mechanisms, and role in the pathogenesis of obesity. Obesity 18, 221–229. 10.1038/oby.2009.228 19644451

[B44] Palma V. Lim D. A. Dahmane N. Sánchez P. Brionne T. C. Herzberg C. D.. (2004). Sonic hedgehog controls stem cell behavior in the postnatal and adult brain. Development 132, 335–344. 10.1242/dev.01567 15604099 PMC1431583

[B45] Palouzier-Paulignan B. Lacroix M. C. Aimé P. Baly C. Caillol M. Congar P.. (2012). Olfaction under metabolic influences. Chem. Senses 37, 769–797. 10.1093/chemse/bjs059 22832483 PMC3529618

[B46] Park H. K. Ahima R. S. (2014). Leptin signaling. F1000Prime Rep. 6:73. 10.12703/P6-73 25343030 PMC4166933

[B47] Park H. R. Park M. Choi J. Park K. Y. Chung H. Y. Lee J. (2010). A high-fat diet impairs neurogenesis: involvement of lipid peroxidation and brain-derived neurotrophic factor. Neurosci. Lett. 482, 235–239. 10.1016/j.neulet.2010.07.046 20670674

[B48] Pestell R. G. (2013). New roles of cyclin D1. Am. J. Pathol. 183, 3–9. 10.1016/j.ajpath.2013.03.001 23790801 PMC3702737

[B49] Pierce A. A. Xu A. W. (2010). De novo neurogenesis in adult hypothalamus as a compensatory mechanism to regulate energy balance. J. Neurosci. 30, 723–730. 10.1523/JNEUROSCI.2479-09.2010 20071537 PMC3080014

[B50] Pinto S. Roseberry A. G. Liu H. Diano S. Shanabrough M. Cai X.. (2004). Rapid rewiring of arcuate nucleus feeding circuits by leptin. Science 304, 110–115. 10.1126/science.1089459 15064421

[B51] Rahmouni K. Sigmund C. D. Haynes W. G. Mark A. L. (2009). Hypothalamic ERK mediates the anorectic and thermogenic sympathetic effects of leptin. Diabetes 58, 536–542. 10.2337/db08-0822 19066310 PMC2646051

[B52] Ramos-Rodriguez J. J. Molina-Gil S. Ortiz-Barajas O. Jimenez-Palomares M. Perdomo G. Cozar-Castellano I.. (2014). Central proliferation and neurogenesis is impaired in type 2 diabetes and prediabetes animal models. PLoS ONE 9:e89229. 10.1371/journal.pone.0089229 24586614 PMC3930705

[B53] Rasmussen B. A. Breen D. M. Duca F. A. Côté C. D. Filippi B. M. Lam T. K. T. (2014). Jejunal leptin-PI3K signaling lowers glucose production. Cell Metab. 19, 155–161. 10.1016/j.cmet.2013.11.014 24361011

[B54] Rehman G. Shehzad A. Khan A. L. Hamayun M. (2014). Role of AMP-activated protein kinase in cancer therapy. Arch. Pharm. 347, 457–468. 10.1002/ardp.201300402 24677093

[B55] Rosa A. I. Gonçcalves J. Cortes L. Bernardino L. Malva J. O. Agasse F. (2010). The angiogenic factor angiopoietin-1 is a proneurogenic peptide on subventricular zone stem/progenitor cells. J. Neurosci. 30, 4573–4584. 10.1523/JNEUROSCI.5597-09.2010 20357108 PMC6632326

[B56] Scholzen T. Gerdes J. (2000). The Ki-67 protein: from the known and the unknown. J. Cell. Physiol. 182, 311–322. 10.1002/(SICI)1097-4652(200003)182:3<311::AID-JCP1>3.0.CO;2-9 10653597

[B57] Sherr C. J. Roberts J. M. (2004). Living with or without cyclins and cyclin-dependent kinases. Genes Dev. 18, 2699–2711. 10.1101/gad.1256504 15545627

[B58] Speisman R. B. Kumar A. Rani A. Foster T. C. (2013). Daily exercise improves memory, stimulates hippocampal neurogenesis and modulates immune and neuroimmune cytokines in aging rats. Brain Behav. Immun. 28, 25–43. 10.1016/j.bbi.2012.09.013 23078985 PMC3545095

[B59] Sultan S. Mandairon N. Kermen F. Garcia S. Sacquet J. Didier A. (2010). Learning-dependent neurogenesis in the olfactory bulb determines long-term olfactory memory. FASEB J. 24, 2355–2363. 10.1096/fj.09-151456 20215526

[B60] Tang B. L. (2008). Leptin as a neuroprotective agent. Biochem. Biophys. Res. Commun. 368, 181–185. 10.1016/j.bbrc.2008.01.063 18222172

[B61] Timsit S. Rivera S. Ouaghi P. Guischard F. Tremblay E. Ben-Ari Y.. (1999). Increased cyclin D1 in vulnerable neurons in the hippocampus after ischemia and epilepsy: a modulator of *in vivo* programmed cell death? Eur. J. Neurosci. 11, 263–278. 9987030 10.1046/j.1460-9568.1999.00434.x

[B62] Villanueva E. C. Myers M. G. Jr. (2008). Leptin receptor signaling and the regulation of mammalian physiology. Int. J. Obes. 32(Suppl. 7), S8–S12. 10.1038/ijo.2008.232 19136996 PMC2648306

[B63] Zhang F. Proenca R. Maffei M. Barone M. Leopold L. Friedman J. M. (1994). Positional cloning of the mouse obese gene and its human homologue. Nature 372, 425–432. 10.1038/372425a0 7984236

